# Investigation of Polymer Aging Mechanisms Using Molecular Simulations: A Review

**DOI:** 10.3390/polym15081928

**Published:** 2023-04-18

**Authors:** Fan Zhang, Rui Yang, Diannan Lu

**Affiliations:** Department of Chemical Engineering, Tsinghua University, Beijing 100084, China; zhangf19@mails.tsinghua.edu.cn

**Keywords:** polymer aging, molecular simulation, aging mechanism, ReaxFF, insulation failure, composite materials

## Abstract

Aging has a serious impact on the properties of functional polymers. Therefore, it is necessary to study the aging mechanism to prolong the service and storage life of polymer-based devices and materials. Due to the limitations of traditional experimental methods, more and more studies have adopted molecular simulations to analyze the intrinsic mechanisms of aging. In this paper, recent advances in molecular simulations of the aging of polymers and their composites are reviewed. The characteristics and applications of commonly used simulation methods in the study of the aging mechanisms (traditional molecular dynamics simulation, quantum mechanics, and reactive molecular dynamics simulation) are outlined. The current simulation research progress of physical aging, aging under mechanical stress, thermal aging, hydrothermal aging, thermo-oxidative aging, electric aging, aging under high-energy particle impact, and radiation aging is introduced in detail. Finally, the current research status of the aging simulations of polymers and their composites is summarized, and the future development trend has been prospected.

## 1. Introduction

Polymer materials have various properties superior to traditional materials; they can not only be used as structural materials but also have a broad development prospect as functional materials [1,2]. However, polymer materials are easily affected by heat, oxygen, water, light, chemical mediators, and other factors. Under the influence of these factors, the chemical composition and structure of the polymer materials change, and their physical properties deteriorate accordingly. This phenomenon is called the aging of polymer materials. Aging failure has become one of the key problems limiting the development and application of polymer materials, which will cause a waste of resources, economic losses, and even environmental pollution. Therefore, in order to take better protective measures to slow down aging and ultimately prolong the service life of materials, it is necessary to study the aging mechanism of materials under specific environmental conditions.

The aging of polymer materials has been studied for many decades, and so far, outstanding progress has been made in experiments, theories, and simulations. Since the degradation of polystyrene was first studied in 1935 [3], in the following decades, researchers have systematically studied the aging and stabilization of polymers [2] using experimental methods. Thermo-oxidative aging [4,5,6,7], photo-oxidative aging [8,9,10], aging under the influence of chemical mediators [11,12,13], and physical aging [14,15] of materials have gradually become hot topics. During this process, theories of aging were also growing. As for the reaction theory of aging, studies showed that polymer materials mainly follow the free radical chain reaction process under the joint action of light, heat, and oxygen [2]; the initiation process is normally considered to be due to the homolysis of C-C or C-H bonds in the molecule, or the reaction of O_2_ with the polymer to generate free radicals [5]. In addition, Day et al. believed that the addition of impurities with initiation effects and anti-aging agents would also have a significant impact on the aging reaction [4]. As for the diffusion theory of aging, Troev et al. found that only when the permeating molecules diffused into the solid polymer could they interact with the polymer [13]. There are currently two theories on the diffusion mechanism, namely (1) the Thermal Energy Fluctuation Theory [16], which proposes that polymer fragments absorb energy, loosen, and rearrange, thereby pushing permeating molecules to new positions and (2) the Free Volume Theory [2], which suggests that permeating molecules can migrate by jumping between free volumes or holes formed in polymer materials. From the end of the 1990s to the beginning of the 21st century, the research gradually shifted from the macro level to the micro level. With the rapid development of computer technology, molecular simulation methods have become as important as experimental methods. Among the many simulation studies on polymer aging, according to different time and space scales, the common molecular simulation methods at the molecular level include quantum mechanics (QM), molecular mechanics (MM), molecular dynamics (MD), Monte Carlo method (MC), and reactive molecular dynamics (RMD) [17,18,19]. In recent years, our research groups has also conducted relevant experimental studies [20,21,22,23,24,25,26,27] and simulation studies [28].

According to the aging mechanism, the main types of polymer aging are physical aging and chemical aging. Physical aging [2] refers to the reversible changes in secondary bonds caused by physical actions on materials. On the other hand, chemical aging [2] involves the breakage of primary bonds, leading to irreversible chemical reactions that alter the molecular structure of the materials. Under the influence of complex factors, physical and chemical aging often occur in combination. Experimental studies have made remarkable contributions to the exploration of the aging mechanism; however, there are also challenges: (1) the aging process is time-consuming; (2) the complex chemical reactions are difficult to analyze in detail from the macroscopic scale; (3) the macroscopic influence of various environmental factors on materials is easy to analyze, but the microscopic influence is difficult to grasp. At present, the understanding of the aging mechanism is far from being complete. With the help of computer simulation technology, various physical and chemical changes of polymers within nanoseconds can be captured and analyzed in more detail, and more breakthroughs at the micro level are expected to appear.

As computer simulation technology plays an increasingly important role in the research of aging mechanisms, many important results have been obtained. In the following section, the commonly used molecular simulation methods in polymer aging research are briefly introduced. Then, the recent progress in simulation research of aging mechanisms under different influencing factors is elaborated separately. In the final section, we summarize the current status and provide prospects for the future trend of aging simulation research.

## 2. The Commonly Used Molecular Simulation Methods

**Figure 1:**
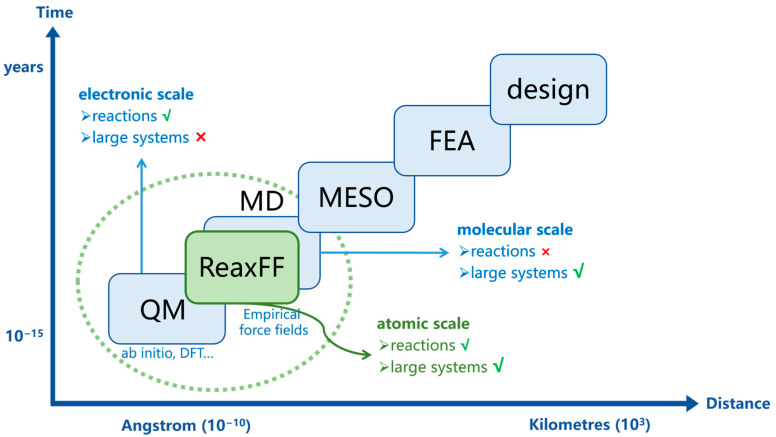
Temporal and spatial scales of different computational chemistry methods, reproduced with permission from van Duin, A.C.T. [30].

Molecular simulation is a technology that uses computers and basic principles to establish molecular models at the atomic level, calculate reasonable molecular structures and behaviors, and then simulate various physical and chemical properties of molecular systems [29]. Since Schrodinger proposed the wave equation and established quantum mechanics in 1926, molecular simulation technology has been developing continuously. At present, in the field of polymer aging research, the most commonly used molecular simulation methods are the traditional molecular dynamics (MD) simulation, the quantum mechanics (QM) method, and the reactive molecular dynamics (RMD) simulation based on the Reactive Force Field (ReaxFF). The temporal and spatial scales of these three molecular simulation methods and other computational chemistry methods are shown in Figure 1. Simulation methods at different scales are applicable to different research ranges of aging; Table 1 provides a general description.

### 2.1. Traditional Molecular Dynamics (MD) Simulation

The traditional molecular dynamics (MD) simulation is one of the most commonly used simulation methods. Based on the classical Newtonian equation of motion, it can solve the motion trajectory of each atom or molecule in the system, and then calculate various properties of the macroscopic system by using the statistical mechanic’s principle [47]. In the MD simulation, the bond connection between atoms is fixed, which ensures high efficiency and accuracy for the study of large systems like polymer systems.

At present, the aging simulation studies using the MD method often focus on intermolecular interactions such as adsorption and diffusion, as well as thermodynamic and kinetic properties under specific conditions [19]. For example, the coarse-grained models are often constructed to study the physical aging of glassy polymers [37,38,39] or changes in polymer properties caused by stress application [37,40,41]; some properties related to the electric field are analyzed [42,43], such as energy, dipole moment, polarizability, and charge distribution; the physical diffusion process of oxygen and water molecules in materials is also investigated [44,45,46]. However, aging is a process involving complex chemical reactions. The traditional MD method cannot calculate the free breakage and formation of chemical bonds among atoms of the system, so it cannot effectively reveal the chemical reaction process at the atomic level. In addition, the construction of complex polymer models, such as crosslinked epoxy resins [48,49,50], is often achieved by the traditional MD method.

### 2.2. Quantum Mechanics (QM)

The quantum mechanics (QM) method takes the electron as the most basic unit and uses the principles of the quantum mechanics to solve problems; the results obtained by the QM method are often very close to the experimental data. Ab initio is one of the QM methods [51], and it can solve the Schrodinger equation without any empirical parameters, but it is too computationally expensive for polymer systems. Another more widely used QM method is density functional theory (DFT) [52,53], a semi-empirical algorithm. This method can provide accurate molecular geometric configuration and electron distribution information, but it can generally only handle systems with less than 1000 electrons. 

For a complex polymer system, it is difficult to study the dynamic behavior, thermodynamic performance, and other overall characteristics of the system using only the QM method [19]. At present, the application of the QM method in polymer aging simulations is often aimed at some simplified systems, local structures, and key chemical bonds to analyze the bond-breaking difficulty and the reaction activation energy [31,32]. Therefore, the QM method can help us further explain the phenomena observed in the aging process, such as the promotion of certain small molecules to the aging process, the formation mechanism of certain products, and the difficulty of certain chemical reactions [28].

### 2.3. Reactive Molecular Dynamics (RMD) Simulation

As can be seen from Figure 1, the simulation scale of the traditional MD method is the molecular scale; the MD method can study large systems, but cannot dynamically analyze the chemical reactions during the aging process. Although the QM method can provide valuable theoretical guidance from the electronic scale, the computational cost is too high for the polymer system. The emergence of reactive molecular dynamics (RMD) simulations, a method on the atomic scale, has made it possible to study the chemical reaction process in large systems. The Reactive Force Field (ReaxFF) [54] proposed by Van Duin in 2001 is the most widely used reactive force field at present. Unless otherwise specified, the RMD methods mentioned in this article all use the ReaxFF.

In the ReaxFF, there is no clear connection relationship between atoms, so the bond order between different atoms should be calculated in real time to judge the connection type between atoms. Since there is no fixed connection between atoms, the process of bond breaking and bond forming exists in the ReaxFF. Therefore, the chemical reaction process can be described. In the ReaxFF, the bond order is a function of the distance between two atoms, and the formula for the uncorrected bond order [19,54] is given in Equation (1).

(1) $${BO}_{ij}^{\prime }=\exp\left[p_{bo1}∙{\left(\frac{r_{ij}}{r_{0}^{\sigma }}\right)}^{p_{bo2}}\right]+\exp\left[p_{bo3}∙{\left(\frac{r_{ij}}{r_{0}^{\pi }}\right)}^{p_{bo4}}\right]+\exp\left[p_{bo5}∙{\left(\frac{r_{ij}}{r_{0}^{\pi \pi }}\right)}^{p_{bo6}}\right]$$

In Equation (1), $r_{ij}$ represents the interatomic distance, $r_{0}^{\sigma }$, $r_{0}^{\pi }$, $r_{0}^{\pi \pi }$ represent the atom parameters, $p_{bo1}$ to $p_{bo6}$ represent the bond parameters; on this basis, the bond order is corrected, however, the specific correction process is not discussed in detail here. 

The expression of the potential energy [54] is given in Equation (2), which is a function of the bond order except for non-bond interactions.

(2) $$E_{system}=E_{bond}+E_{over}+E_{under}+E_{val}+E_{pen}+E_{tors}+E_{conj}+E_{vdWaals}+E_{Coulomb}$$

In Equation (2), $E_{system}$ represents the total system energy, including the bond energy $E_{bond}$, over-coordination energy $E_{over}$, under-coordination energy $E_{under}$, valence angle energy $E_{val}$, penalty energy $E_{pen}$, torsion angle energy $E_{tors}$, conjugation energy $E_{conj}$, non-bond van der Waals energy $E_{vdWaals}$, and non-bond Coulomb energy $E_{Coulomb}$.

Since ReaxFF does not describe the chemical reaction by describing the electron transfer behavior, the calculation cost of RMD is greatly reduced compared with the QM method. The force field parameters of ReaxFF are obtained by fitting the training set generated by the first principle calculation. Therefore, when describing the energy and transition state of chemical reactions, the results from the ReaxFF and QM are in relatively good agreement [54]. The computational requirement of RMD is between that of QM and MD. At present, the RMD method can probably handle systems containing millions of atoms [55], and the simulation time can reach the order of 100 nanoseconds [56], so it has been widely used in polymer systems. The extensibility and portability of the RMD method are very strong, and the types and fields of elements that can be studied are constantly expanding [57,58]. 

In aging studies using the RMD method, the dynamic process of chemical reactions can be obtained. The research content focuses on analyzing the dynamic changes of the components and numbers of reactants/products in the reaction process, analyzing the reaction path, generation mechanism, and the reaction rate of specific substances [33,34,35,36]. Therefore, we can obtain detailed microscopic insight into the chemical aging inside the materials and construct the aging reaction network.

## 3. Studies on Polymer Aging Mechanisms

**Figure 2:**
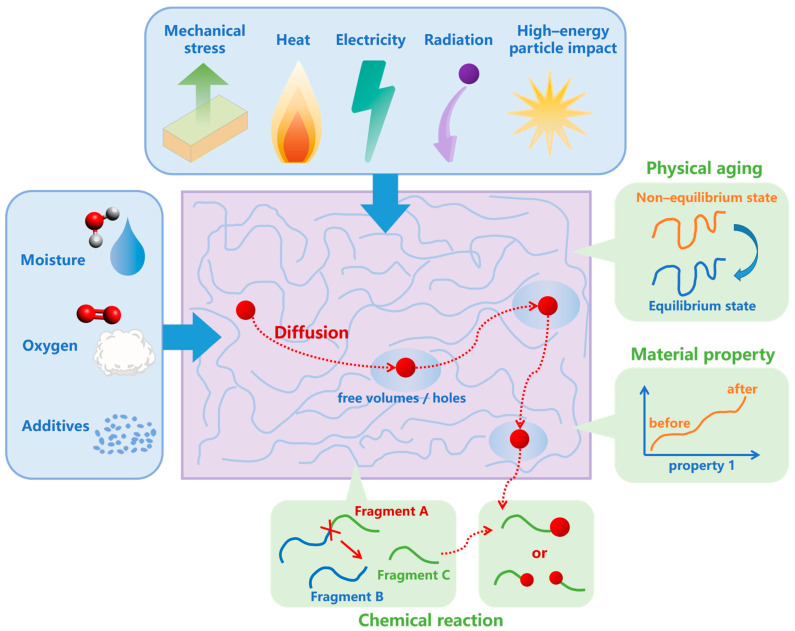
An overview of aging mechanisms in the current aging simulation research.

Aging is related to external environmental factors, as well as internal chemical composition, internal structure, and molecular weight distribution [59,60,61,62]; the aging process involves complex physical and chemical changes. Figure 2 shows an overview of the aging mechanisms used in the current aging simulation research: under the influence of stress, heat, oxygen, water, electricity, high-energy particle impact, radiation and additives, physical aging, diffusion (such as jump diffusion), chemical reactions, and other behaviors that occur inside the materials. The macroscopic influence of these behaviors can be reflected in the change of some properties of the materials.

**Figure 3:**
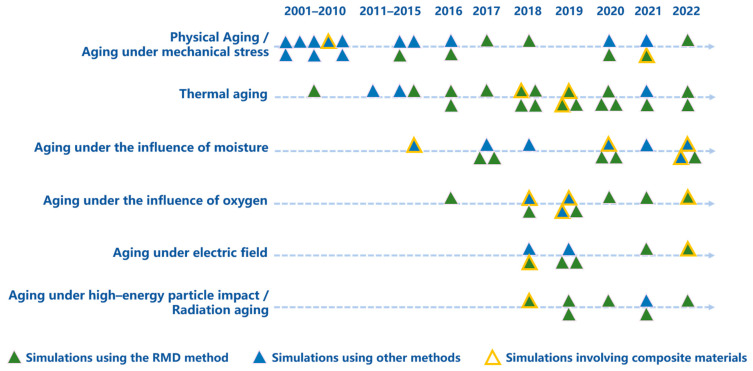
A summary of the aging simulation works on polymers and their composites under different in-fluence factors since the 21st century. In this figure, simulations using the RMD method are rep-resented by green triangles, simulations using other methods are represented by blue triangles, and simulations involving composite materials are marked with yellow edges.

As shown in Figure 3, since the 21st century, physical aging and aging under mechanical stress were the first to be widely studied, then thermal aging became the main research focus. In the last ten years, on the basis of thermal aging, the effect of moisture and oxygen was gradually taken into account. In the last five years, the research scope of aging simulations continued to expand, and now involves the aging influence factors of the electric field, high-energy particle impact, and radiation.

### 3.1. Physical Aging and Aging under Mechanical Stress

In the early stage, the simulation work on aging focused on physical aging. The condensed state structure of glassy polymer transforms from a non-equilibrium state to an equilibrium state through the Brownian motion of the chain segment in a small area; this relaxation process is called physical aging. Physical aging can change the mechanical properties, thermal properties, and dielectric properties of materials. For the specific material, during its physical aging process, a reversible secondary bond change occurs [2] and its condensed state structure is altered [63,64], but its molecular structure is not changed.

In the study of physical aging, the coarse-grained models are often used: Andrejew et al. [65] used a coarse-grained lattice model to simulate the glassy polymer melt, and the MC simulation results showed that the model exhibited obvious physical aging effects close to the glass transition. Dokholyan et al. [39] built a “beads on a string” model and employed a discrete molecular dynamics algorithm; they proved that for the dynamics of a long collapsed polymer chain, aging was an intrinsic feature. Liu et al. [66] constructed a coarse-grained polymer nanocomposite model and used MD simulations to analyze the impact of nanoparticles on physical aging. Some studies focused on the effects of physical aging. With the support of the MC and MD method, Wang et al. [67] found that physical aging decreased the permeability and increased the permeability selectivity of poly(1-trimethylsilyl-1-propyne) (PTMSP) membranes. Yiapanis et al. [68] conducted a classical MD study and showed that aging increased adhesion to carbon particles of the polyester surface compared to the initial rigid polymers. Physical aging is also affected by various factors. Frisch et al. [69] analyzed the effects of the solvent on the polymer relaxation; they used different numerical methods (MC+MD) and showed that the aging effect almost disappeared in the presence of a solvent. Tang et al. [70] found that when the system of polymer films was confined at extremely small thicknesses, the physical aging of the films would be suppressed; they conducted their research through the MC method. In a subsequent study, they included the free volume diffusion and annihilation (FVDA) model into the MC simulation to further explore the physical aging behaviors of ultrathin polymer films [71]. 

When stress is applied to polymer materials, the physical aging of materials is mainly induced. Several traditional MD simulation works have studied the physical aging of materials under uniaxial tensile [37,38,72], uniaxial compression [38,73], and shear [40] deformation. When the material is stressed, the microstructure of the polymer chain changes, which also enables the mechanical energy accepted by the system to be dissipated by relaxation. However, when the applied stress exceeds a certain limit, the relaxation can no longer completely consume the mechanical energy, leading to the fracture of the material [74].

When studying the mechanical response or failure mode of polymers through the RMD simulation, epoxy resins are the most commonly studied materials. Generally, the model is established in the classical force field first. Koo et al. [75] captured brittle failure in epoxy-based thermoset polymers using ReaxFF. The molecular vibration due to temperature re-equilibrates the elongated covalent bonds thus preventing the bond from breaking. Therefore, they developed an ultra-high strain rate (UHSR ≈ 10^13^ s^−1^, higher than molecular vibration frequency) approach to decouple the thermal vibration from the deformation test. They calculated the bond dissociation energy (BDE) of a specific C-C bond, to describe the extent of damage to the systems under fracture analysis. Odegard et al. [76] demonstrated that the elastic properties and yield point of crosslinked epoxies could be reliably predicted using the ReaxFF with the Liu parameter set [77]; they suggested that when comparing predicted elastic properties with experimental results, the effect of different strain rates should be taken into account. Later, they compared the mechanical properties of di-, tri-, and tetra-functional resin epoxies [78]. Considering not only the resin function, they also defined the monomeric degree index (MDI) as the average number of monomeric units covalently bonded to a given monomeric unit. The MDI, which includes the combined effect of functionality and crosslink density, has a greater effect on Young’s modulus and yield stress. Furthermore, they chose pristine graphene nanoplatelets (GNPs), highly concentrated graphene oxide (GO), and functionalized graphene oxide (FGO) to reinforce the epoxy resin [79]. After predicting the mechanical properties of these composites, they found that the introduction of oxygen and functional groups turned the sp^2^ structure of GNP into the more compliant sp^3^ structure, leading to a decline in the in-plane shear module and elastic Young’s module, but then significantly improved the interlocking mechanism with the hosting matrix and the interfacial interaction energy. Chowdhury et al. [48] extended their study to a larger strain range, and analyzed the failure mechanism of epoxy systems with different degrees of cure and cross-linker. They found that a higher degree of cure and lower molecular weight (MW) of the cross-linker produced a higher modulus and strength; however, a lower MW of the cross-linker also led to lower strain to failure and lower absorbed energy. 

In addition to these studies on epoxy resins, Yilmaz et al. [80] constructed poly(p-phenylene terephthalamide) (PPTA) fibers that had real size and structure, which were used to conduct RMD simulations. They proposed an empirical formula to predict the tensile modulus of a PPTA fiber with specific crystallinity and defect density. They also observed domains in the crystalline region of fibers; the failure of fibers was caused by the breaking of chains located at these domain boundaries. Zhang et al. [81] found that the cross-linking degree has a great influence on the mechanical properties of styrene–butadiene rubber (SBR) through an RMD approach; when the bond energy and bond angle energy associated with a cross-link increase, the total energy of the system increases distinctly, so a higher cross-linking degree was associated with higher tensile stress. Furthermore, Fiorin et al. [41] studied the shear response of ideal poly(p-phenylene terephthalamide) crystals through MD and QM methods.

**Figure 4:**
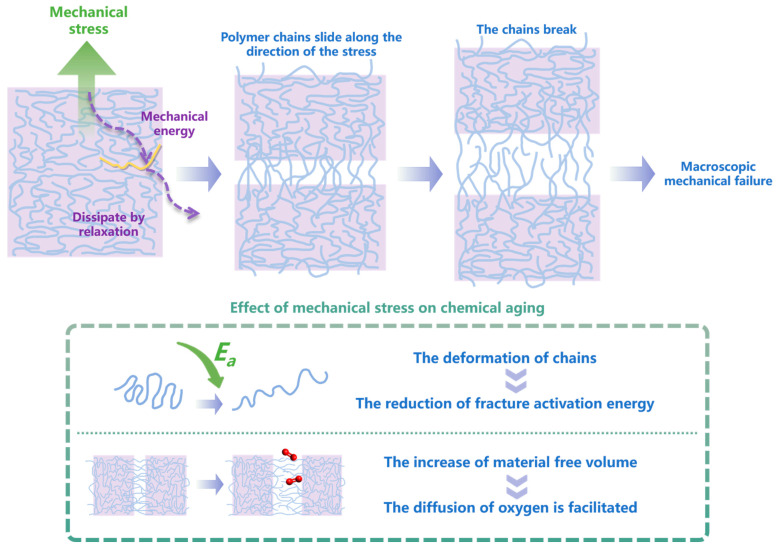
The aging mechanisms under mechanical stress (tensile stress used as an example).

Zhu et al. [74] pointed out that chemical aging did not occur when stress was the only factor. However, stress can affect the chemical aging of materials by affecting the following two aspects: (1) the activation energy of chain breaking and (2) oxygen diffusion. As can be seen from the above, there are not enough simulation studies on the effects of stress on chemical aging, and the advantages of the RMD method have not been fully exploited. Figure 4 summarizes the main mechanisms of aging under mechanical stress, using tensile stress as an example.

### 3.2. Thermal Aging

**Figure 5:**
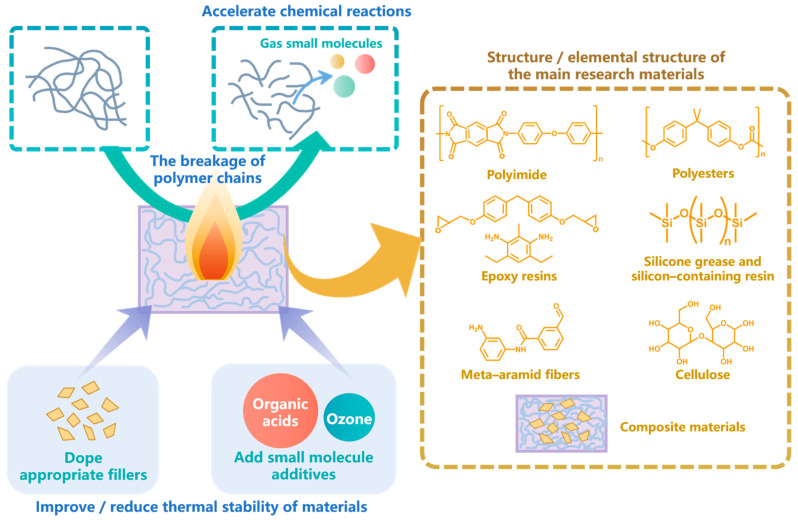
The main mechanism and the main research materials for thermal aging.

Thermal aging is the main direction in the aging research field, which is mainly the result of the further chemical reaction of materials promoted by temperature rise [1]. Since the 2010s, thermal aging has become the most important aspect of aging simulation research. For thermal aging, the RMD method, which can analyze the dynamic process of chemical reactions, is the most commonly used simulation method, and the DFT of QM is also a common method. Many different types of polymer materials have become the research objects for thermal aging. The main mechanism and the main research materials of thermal aging are summarized in Figure 5.

For polyimide (PI): Based on the DFT of QM, Luo et al. [82] analyzed the thermal aging mechanism of PI with five monomers; they found that the ether bond (C-O-C) on the main chain of PI and the C-N bond on the imide ring were easy to break under heated conditions. Lu et al. [83,84] used a PI with four monomers as an example and conducted RMD simulations; their results shed further light on the aging mechanisms of PI. They showed that the C-N bond and ether bond on the main chain of the PI decreased its polymerization, and the breakage of the C-N bond on the imide ring promoted the formation of small molecule products (CO_2_ and CN); ultimately, these chemical changes led to the insulation failure of PI at high temperatures.

For polyesters: Saha et al. [85] carried out an experimental study and RMD simulation simultaneously, aiming to understand the thermal decomposition characteristics of polyacrylicester terpolymer. Consistent with experimental observations, alkenes, carbon dioxide, and alkyl radicals were detected as the products in the RMD. Based on the RMD results, the evolution mechanism of the products was analyzed in detail. Zhao et al. [86] employed the ReaxFF in their simulation to find the thermal aging mechanisms of polycarbonate (PC). The results showed two main pathways for the breakage of the PC main chain: (1) the breakage of the terminal group and (2) the breakage between PC monomers. The activation energy and the pre-exponential factor in the RMD simulation were also calculated, which were in agreement with the experimental results. 

For epoxy resins: To obtain the aging mechanism of insulating epoxy resins caused by high temperatures, Zhang et al. [87] used ReaxFF to investigate the decomposition process of epoxy resins cured by anhydride. They found the initiation of the aging reaction was the cleavage of an ester bond linked with an epoxy resin; during the aging process, the following small molecule gases were produced sequentially: CO_2_, CH_2_O, CO, and H_2_O. They also explored the thermal aging properties of epoxy resins in a SF_6_/N_2_ insulation mixture [88] and after simulation in the ReaxFF, they further verified the following conclusion: The CO_2_ and H_2_O produced during the aging process of the epoxy resin could intensify the degradation of the dielectric property of SF_6_. Chi et al. [89] combined the bisphenol F epoxy resin and some discharge active products (nitric acid and ozone) together, and under the action of the active products, they found that the thermal stability and the initial dissociation temperature of epoxy resin decreased. 

For silicone grease and silicon-containing resins: Chenoweth et al. [35] expanded the ReaxFF to describe carbon–silicon systems, which they used to investigate the thermal aging of the polydimethylsiloxane polymer (PDMS). Through tracking the generation rate of the typical product methane, they studied the temperature and pressure dependence of PDMS decomposition. Then, they added several additives (water, SiO_2_ slab, ozone, NO) into the system, and learned how they affected the PDMS stability. Wei et al. [90] also studied the thermal aging of PDMS using RMD; at the end of their paper, they considered the effect of oxygen on the thermal aging process, which we will discuss in detail in Section 3.4. Zheng et al. [91] analyzed the pyrolysis behavior of silicon-containing arylacetylene resin (PSA); both experimental and RMD simulation results showed that CH_4_ and H_2_ were the main gas products, and the molecular reaction trajectory indicated their main formation pathways were hydrogen abstraction reactions.

For meta-aramid (PMIA) fibers: Yin et al. [92] studied the thermal decomposition mechanism of PMIA fiber using the RMD method. The results indicated that the C_aromatic ring_–N and C=O in PMIA fiber elements were easy to decompose first. At higher temperatures, the composition of products produced by the thermal aging of PMIA was more complex. Hydrocyanic acid, which was produced at high temperatures, could be used to detect thermal faults in PMIA. In another work they published that same year [93], the effects of small organic acids were studied, and formic acid was proved to accelerate the thermal aging of PMIA.

For cellulose: Zhang et al. [94] studied the generation mechanism of methanol from cellulosic insulating paper during the thermal aging process by simulating the pyrolysis of cellobiose through RMD simulation. They also studied the generation mechanism of ethanol [95]. Because this molecular system incorporated hydrogen bonds, in order to improve the accuracy and effectiveness of the calculations, they mixed force-bias Monte Carlo (fbMC) into ReaxFF. The generation pathways of CO_2_ and CO were also analyzed. Zheng et al. [96] analyzed and tracked CO_2_ and CO by RMD simulation; these reaction pathways were given a lot of attention because the CO_2_/CO ratio could reflect the aging state of cellulosic insulating paper. Hao et al. [97] simulated the internal aging mechanism of natural ester delaying the aging of the cellulose insulation polymer in their MD simulation, using the polymer consistent force field (PCFF). They found that natural ester could form hydrogen bonds with hydronium ions, which led to fewer hydronium ions in the cellulose and finally reduced the acid hydrolysis reaction of cellulose. 

There is also a small amount of work focusing on composite materials. Pal et al. [98] studied the effect of nano-reinforced materials on the mechanical properties of polyurea–polyhedral oligomeric silsesquioxane (POSS) nanocomposites through MD simulation. Saha et al. [99] chose the oligomeric polyacrylicester (ACM) chains as the polymer matrix; they found that the nanosilica reinforcement could decrease the rate of the degradation of ACM nanocomposite. Their conclusions were backed up by experiments and RMD simulations. Similarly, they also introduced a model graphene-oxide (GO) particle into the ACM system [100], and introduced nanosilica into a hydrogenated acrylonitrile–butadiene rubber model [101]. They used experiments and RMD simulations to better understand the effect of nanometer fillers on the thermal stability of polymer materials. However, the microscopic mechanism at the interface needs to be further explored. Du et al. [59] used the QM method and calculated the chemical bond energy of the HTPB (hydroxyl-terminated polybutadiene)–TDI (toluene diisocyanate) simplified adhesive system; they also pointed out that the oxidation linking reaction was the main cause of HTPB propellant aging due to the small reaction activation energy. However, that work only focused on the adhesion agent of the propellant. In an MD simulation work by Kong et al. [102], the aging degradation effect of adhesion agents on the diffusibility and compatibility of energetic plasticizers was studied. 

### 3.3. Aging under the Influence of Moisture

Moisture is an important environmental factor, since the presence of moisture will change the structure, properties, and aging behavior of materials. Kwon et al. [103] analyzed the hygroelastic behavior of thermosetting epoxy, and several properties such as the diffusion coefficient of water and the coefficient of moisture expansion were predicted with the help of MD methods. Arash et al. [36] studied the aging mechanism of polyamide 6,6; in addition to the RMD method, they also employed a collective variable-driven hyperdynamics (CVHD) method [104] in order to accelerate the simulation. As shown in their calculation results, when the water content increased, the activation energy of thermal degradation decreased, while the pre-exponential factor increased.

When studying the influence of moisture on aging, the factor of temperature is often taken into account because materials usually tend to age under the combined action of moisture and temperature when they are used and stored; this aging process is called hygrothermal aging. During the hygrothermal aging process of polymers, water molecules affect the stability of polymers by forming hydrogen bonds with the polymers or participating in reactions. Lin et al. [105] studied the effect of moisture absorption on the structure and properties of polyimide (PI) films using experiments and an MD simulation. As the moisture content increased, the mobility of the molecular chains increased, which indicated higher ductility of the PI film; this change was mainly attributed to the hydrogen bond between H_2_O and polymer chains. Karuth et al. [106] simulated the hydrothermal aging of crosslinked epoxy polymers through an RMD simulation. In the crosslinked epoxy polymer network, they observed the protonation of the water molecule and the nucleophilic attack on the C-O bond of the ether linkages. They also pointed out that the spatial arrangement and steric hindrance of the network would affect the selectivity of water molecules for the hydrolysis reaction in the network, thus affecting the ability of the network to resist moisture. Zhao et al. [107] took cellobiose, the structural unit of the cellulose chain of insulating paper, as the research model. Through RMD simulation, it was found that a large amount of water would be generated in the process of cellobiose pyrolysis. These water molecules moved in the free spaces of cellulose chains, and bonded with hydroxyl or ether oxygen atoms on cellulose chains to form hydrogen bonds and thus, the hydrogen bond network of cellulose was destroyed and the cross-linking degree and mechanical strength of cellulose were decreased, which finally led to a lower anti-aging ability. Li et al. [108] also proved that water molecules could easily form hydrogen bonds with cellobiose molecules at high temperatures, thus destroying the structure of cellobiose. Zeng et al. [109] studied the thermal aging of polydimethylsiloxane (PDMS) using the ReaxFF, and found that water promoted the cleavage of PDMS, and the decomposition of water provided H and O to the reaction system, which promoted the formation of various products. Besides hydrothermal aging, Kroonblawd et al. [110] built a model copolymer of polydimethylsiloxane and polydiphenylsiloxane, and they studied the chemical degradation pathways in the copolymer following phenyl excitations. Compared to a dry environment, they found that the moisture could significantly affect the reaction pathways and product formation probabilities; they pointed out that there might be a synergistic effect between moisture and radiation. In order to better predict the condensed-phase copolymer chemistry, they used a quantum-based molecular dynamics (QMD) method.

**Figure 6:**
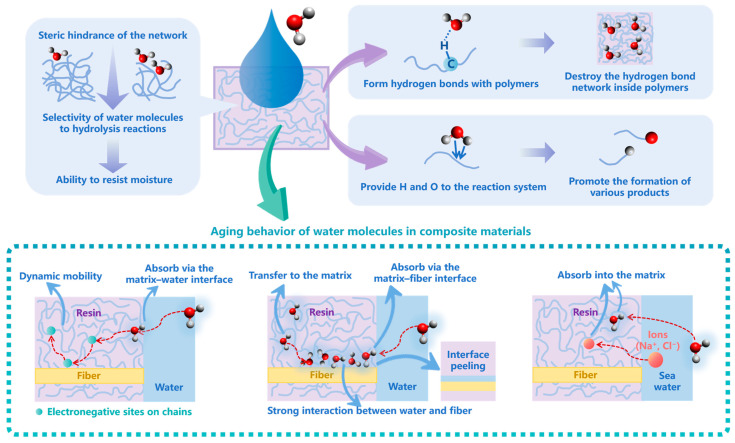
The aging mechanisms under the influence of moisture.

In recent years, how moisture affects the aging of composite materials, especially the aging behavior at the interface, has attracted the attention of researchers. Vuković et al. [46] established a model of a fiber–epoxy composite using the MD method, and analyzed the moisture ingress mechanism at the early stage of the hydrothermal aging process; they found that water was preferentially absorbed via the matrix–water interface, not the fiber–matrix interface. The dynamic mobility of polymer chains facilitated the ingress of water molecules, and the electronegative sites on these chains could capture water molecules, allowing them to penetrate deeper into the composite. The moisture ingress mechanism of the composite was also studied by Li et al. [45]. In their findings, the water molecules were preferentially absorbed in the graphene oxide (GO) sheets–epoxy interface; due to the strong interaction between water and sheets, water molecules were subsequently transferred into the epoxy matrix. Zhang et al. [111] studied the degradation mechanism of the glass fiber-reinforced polymer (GFRP) composites in a seawater environment (the Na^+^, Cl^−^, and water molecules) by MD simulation. The results showed that the ions and water molecules permeated into the resin matrix together, resulting in the swelling of the resin matrix and the degradation of its mechanical properties. Higher seawater content accelerated the movement speed and decreased the concentration of resin matrix on the fiber surface, which finally caused the degradation of the interfacial performance of GFRP. It can be seen that the microscopic mechanism of how water ultimately affects the interface of composite materials still needs to be further analyzed and has vital practical significance. The main aging mechanisms under the influence of moisture obtained in the above works are summarized in Figure 6.

### 3.4. Aging under the Influence of Oxygen

Oxygen is another important environmental factor. The thermo-oxidative aging process has been analyzed in most of the aging simulation studies involving the factor of oxygen. Because the thermo-oxidative aging phenomenon of polymers is extremely common since this process is affected by temperature and oxygen, the aging mechanism is more complex than that of a single aging condition. Huang et al. [112] conducted an RMD simulation on Kapton-type polyimide; through product analysis, they demonstrated that compared to a oxygen-free environment, an oxygen-enriched atmosphere accelerated the aging of polymers. Zhang et al. [34] concluded the reaction paths of oxygen in the thermal decomposition of anhydride-cured epoxy resins using the RMD method, and found that the oxygen could advance the initial break time of the main chain by introducing a carbon–oxygen double bond to the tertiary carbon atoms linked to an oxygen atom. As Sun [113] noted in his research, the oxygen molecules promoted the break of the hydrogenated nitrile rubber (HNBR) chains, and the formaldehyde, acetaldehyde, and other organic compounds were generated, which would weaken the stability of the molecular chains. Zhu et al. [114] used RMD and DFT to study the thermal pyrolysis of polyethylene, and found that the presence of oxygen sped up the aging process and promoted the formation of more chemical defects. In our previous research [28] on the thermo-oxidative aging mechanisms of polypropylene, through the simulation with ReaxFF, we observed that the oxygen molecules could also react with the small molecule aging products to generate active free radicals, which further promoted the aging process.

**Figure 7:**
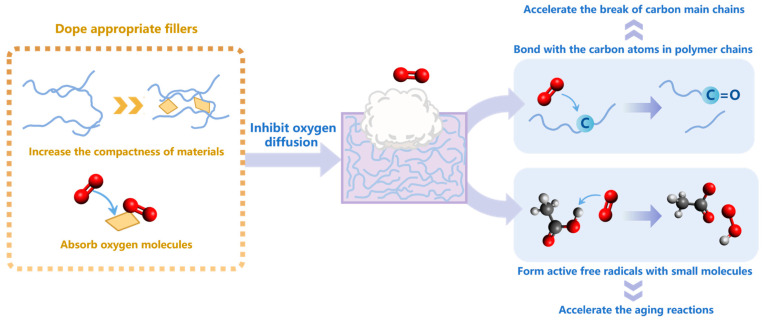
The aging mechanisms under the influence of oxygen.

For composite materials: Zhang et al. [44] studied the diffusion of O_2_ in polyethylene/poly(urea formaldehyde) composites using an MD simulation; it was found that excessive doping of poly(urea formaldehyde) microcapsules into polypropylene would accelerate the diffusion of O_2_, thus accelerating the aging of the composite. In this case, nano-SiO_2_ could be doped into the composite, and the polymer chains were absorbed on the nano-SiO_2_ surface due to hydrogen bond and vdW force, increasing its compactness and inhibiting the diffusion of O_2_. In a paper by Wang et al. [115], the diffusion of O_2_ through different composite systems was studied with the support of RMD and DFT methods. They obtained the BN-C_B_ by substituting carbon atoms for boron atoms on boron nitride nanosheets (BNNSs), and doped the BN-C_B_ into PI. They found that the adsorption effect of BN-C_B_ on O_2_ was significant; by inhibiting the diffusion of O_2_ into PI, the oxidation reaction in the material could be effectively inhibited. Luo et al. modified the original materials by doping solution-polymerized styrene butadiene rubber (SSBR) with antioxidant-functionalized silica (SiO_2_-g-MC) [32], or doping natural rubber (NR) with antioxidant N-isopropyl-N’-phenyl-p-phenylenediamine (4010NA) and silica (SiO_2_) [116] to enhance the resistance of the materials to thermo-oxidative aging; in their work, the experimental method, MD, and QM were combined. The main aging mechanisms under the influence of oxygen obtained from the above works are summarized in Figure 7.

### 3.5. Aging under an Electric Field

The aging of polymer materials is also affected by the external electric field. Li et al. [43] analyzed the influence of the external electric field on the molecular structural property of silane-crosslinked polyethylene (XLPE) cable materials using the quantum chemical simulation method. They found that the C-C bonds and C-H bonds at both ends of the main chain broke preferentially under a high electric field, generating free radicals. At the same time, intramolecular charge transfer occurred, which resulted in the C and H free radicals after bond breaking having a higher charge and forming a space charge. In an MD simulation study conducted by Liang et al. [42], a high-intensity electric field seriously damaged the potential energy of a methyl vinyl silicone rubber, resulting in the contraction and degradation of the molecular structure, and the elastic modulus of the molecular chain also gradually increased. In other words, the high-intensity electric field enhanced the stiffness and brittleness, and reduced the mechanical properties of silicone rubber materials, thus accelerating the aging process.

**Figure 8:**
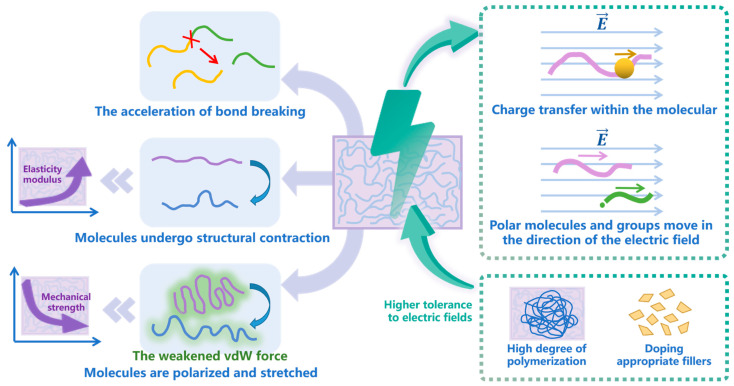
The aging mechanisms under an electric field.

In several RMD simulation studies, Li et al. [117] studied the aging mechanism of PI under an electric field. It was found that the external electric field would change the charge structure, accelerating the breaking of the chemical bond. C-N bonds in the imide ring broke first under an electric field, then the benzene ring broke, precipitating small gas molecules. Ni et al. [118] mentioned that the existence of electrical stress induces the polar molecules and groups to move in the direction of the electric field. However, when both electrical and thermal factors worked together, the temperature effects dominated during the dissociation process. Xin et al. [119] found in their study that polyethylene terephthalate (PET) molecules with a higher degree of polymerization were more tolerant to an electric field. The presence of an electric field induces the PET molecules to become polarized and stretched, and the van der Waals forces within and between chains are weakened, leading to a decrease in the mechanical strength of the PET. The study of Wang et al. [120] also confirmed the weakening effect of the electric field on the van der Waals forces of the polymer system. They also investigated the electric decomposition mechanism of composites [121]; compared with a high concentration of BNNSs, they found that the doping of a low concentration of BNNSs into PI could improve the breakdown strength of the material, and prevent electric distortion caused by excessive ion aggregation. The main aging mechanisms under an electric field obtained from the above works are summarized in Figure 8.

### 3.6. Aging under High-Energy Particle Impact/Radiation Aging

The wide application of polymers and their composites in the aerospace field makes it crucial to study the erosion of ultrafast particles. Materials also tend to be damaged when they are impacted by some high-energy particles. The polymer materials in spacecraft structures are vulnerable to the hypervelocity impact of a large amount of atomic oxygen (AO) in the lower Earth orbit. Ashraf et al. [33] studied the resistance of different epoxy resins to AO using the RMD method, and found that the epoxy resin exhibited higher resistance and lower mass loss when its curing agent contained a stable aromatic ring structure. Yuan et al. [122] analyzed the failure process of poly(vinylidene fluoride) (PVDF) and its composites containing a polyhedral oligomeric silsesquioxanes compound (3,3,3-trifluoropropyl)8Si_8_O_12_ (FP-POSS) under the influence of a single silicon–oxygen cluster. As the RMD simulation showed, the first step of PVDF chain breakage was related to the impact of the cluster. After the impact, most of the kinetic energy of the cluster was transferred to the PVDF chain; then, the chain broke and generated a large number of free atoms and free radicals. In addition, adding FP-POSS to PVDF could significantly improve the erosion resistance of the material. Huang et al. [123] studied the impact effect of DC corona discharge plasma. They found that the incident impact of plasma increased the energy of the PI main chain, which in turn destroyed its stability. Then, the PI chains reacted with plasma, dissociated active free radicals, and finally, released small molecule products.

**Figure 9:**
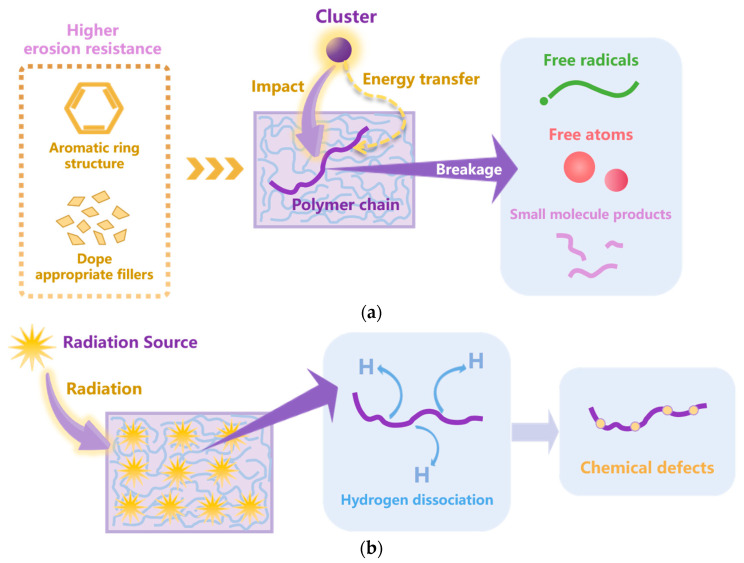
The aging mechanisms under (**a**) high-energy particle impact and (**b**) radiation effect.

In simulation studies of radiation aging, researchers have simulated the radiation process in different ways. Rahnamoun et al. [124] studied the damage to Kapton PI caused by electron beam irradiation. In their RMD simulation, the electron beam model along the PI structure was a string of immobile dummy atoms assigned a zero mass and no charge; when the electron beam was turned on, the negative charge of these particles was turned on for a short time. They found that during the electron beam irradiation, hydrogen atoms first dissociated from different locations in the PI main chain, initiating various subsequent chemical reactions. Li et al. [125] analyzed the damage to polyethylene by beta-decay of substituted tritium. When the polyethylene is exposed to a tritium environment, the tritium–hydrogen exchange will occur, which creates a defect (lack of hydrogen) in the polyethylene chain. Therefore, they removed hydrogen atoms from the polyethylene model to express the decay effect of substituted tritium. The RMD simulation results showed that the loss of hydrogen atoms reduced the order of the polyethylene structure. In a work from Chen et al. [126], the formation of chemical defects in polyethylene was a specialized study. Yeon et al. [127] evaluated the radiation resistance of polystyrene using the RMD method; they transferred the γ-ray energy to the initial velocity of the primary knock-on atom. They found that high γ-ray energy would lead to the chain scission of polystyrene. For the fluorinated polystyrene, the presence of fluorine enhanced the stability against radiation. This is because the charge carrier mobility was increased by the highly ordered π-stacking of fluorinated benzene. Furthermore, Gervais et al. [128] analyzed the radio-oxidation of polyethylene by He ions with the support of an MC simulation. The main aging mechanisms under high-energy particle impacts and radiation effects from the above works are summarized in Figure 9.

## 4. Conclusions and Outlook on Future Work

### 4.1. Conclusions

This review provided an extensive overview of the aging mechanism works on polymers and their composites using molecular simulation methods in the last twenty years. The main conclusions of this review are as follows:

(1) Among the various research methods, the traditional MD simulation method based on classical force fields is mostly applied in the study of mechanical properties of materials, physical aging, diffusion behavior, and other physical processes; it also plays an important role in the construction of complex polymer and composite material model systems. The RMD method, which can study the dynamic processes of chemical reactions in large systems, has become the dominant approach to study the aging mechanism, especially when it involves chemical changes. The QM method is often used as an auxiliary method.
(2) These simulation works have involved various influence factors: internal structure, mechanical stress, heat, oxygen, moisture, electric field, high-energy particle impacts, and radiation. These factors are sometimes studied together, such as moisture and heat, oxygen and heat, electric field and heat, etc. When a variety of factors act together on materials, they tend to affect each other and accelerate the aging process. The physical influences of these factors on material aging are mainly reflected in changing the morphology or movement state of polymer chains, and affecting the diffusion of small molecules or atoms; from the perspective of chemistry, the main effect is the breaking of chemical bonds (the breakage of chains, the removal of small groups or atoms). The starting time of the simulation research on the aging under different influence factors is different, and the depth and breadth of current research are also different. It is worth noting that the experimental research on photoaging started early and plays an important role, but the molecular simulation research on photoaging has been hardly involved so far, which may be due to the difficulty in representing light conditions in molecular simulations.
(3) The research objects of these simulations are varied, and the research contents are relatively scattered. Most of the works are based on the actual storage or use conditions of the studied materials, attempting to explore the aging failure mechanism at the micro level. For each kind of studied material, its aging mechanisms under specific influencing factors has not been explored deeply enough. In addition, compared with pure polymer materials, there are relatively few works that focus on composite materials, and even less attention is paid to the aging characteristics at the interface region. Several studies noted the effects of aging promoters and inhibitors, or the incorporation of other materials into polymers to improve the anti-aging ability.

### 4.2. Outlook on Future Work

The objectives of aging simulation research are exploring aging mechanisms and predicting the life of materials. In order to better achieve the research objectives, aging research in the future will focus on the following points.

#### 4.2.1. Multi-Scale Simulation

In order to fully analyze the aging mechanism, the combination of various simulation methods at different scales will become the choice of more researchers. In addition to the molecular simulation methods (RMD, MD, QM) described above, the Finite Element Analysis (FEA) method has also been applied to the aging simulation of polymers and their composites [129,130,131,132,133]. The results of the FEA simulation can provide a reference for constructing the connection between the experimental and simulation results. Coupling analysis from the atomic scale to the continuum scale can be realized by combining the FEA method with molecular simulation methods [134,135,136]; this multi-scale method is expected to be popularized in the aging research field. In the durability prediction of materials, multi-scale analysis is also a trend. For composite materials, researchers have built various engineering models based on the aging at different scales (micro level, meso level, and macro level) [137], and they are committed to predicting the durability of polymer composites with modelling of their macroscopic properties.

#### 4.2.2. The Aging Database and Machine Learning

In recent years, there have been studies focused on building the aging database. Liu et al. [138] collected over 1900 experimentally determined mechanical properties of FRP materials subjected to long-term environmental effects; the corresponding durability database was also established. However, they also mentioned that the data from different references were highly dispersed and would affect the accuracy of the durability prediction model developed later. They suggested that the standard experimental methods should be improved and unified. For the long-term performance prediction of materials in the environment, there have been several works on the construction of engineering models based on short-term experimental data [137,139,140]. However, with the development of artificial intelligence, machine learning has become a very promising method to predict the life of materials. Some researchers have adopted machine learning methods to develop prediction models related to material aging. For example, Zhang et al. [141] extracted textural features from SEM images of wheat straw/polypropylene composites, and then recognized and classified the SEM images in different aging periods based on an intelligent classifier. Doblies et al. [142] used an artificial neural network (ANN) and Fourier-transform infrared spectroscopy (FTIR) to predict and quantify the aging time, temperature, and residual strength of an epoxy resin. Ghaderi et al. [143] introduced a physics-informed multi-agent constitutive model to describe the effect of single-mechanism chemical aging on the behavior of the cross-linked elastomer. Therefore, the development of lifetime prediction models for polymer materials through artificial intelligence and big data methods will be a promising direction in the subsequent research. 

#### 4.2.3. Explore the Aging Mechanism via Combining Theory and Data-Driven Methods

The data sets that these prediction models require are currently mainly based on experimental data. Through computer simulation, we can also obtain abundant aging data, which contains a large amount of microscopic information that cannot be obtained by experiments. These simulation results can be used to enrich the aging database, and help us generalize a more comprehensive and detailed theory of aging. With these simulation results, the prediction models related to aging in the future will not be limited to predicting the macroscopic properties of materials after a specific aging time, but also the aging mechanism of materials with a specific chemical composition. Therefore, we can expect that the combination of theory and data-driven methods may accelerate the pace of exploring the aging problems.

Last, but not least, with the continuous development of polymer aging simulation methods, our objective will not only be confined to how to improve the stability of materials, but can also be extended to the controllable degradation of materials, which is also an important direction of green chemistry. The design principles of degradable polymer materials can be obtained by studying the influence of internal and external factors on material degradation [118]. At present, there are several simulations that set their research objective as promoting the degradation of materials [119,120,121], which is contrary to the anti-aging research mentioned in this paper. However, the simulation methods adopted by these studies are similar, and both of these research areas are aimed at achieving sustainable development.

## Figures and Tables

**Table 1:** The commonly used molecular simulation methods in polymer aging research.

Method	Scale	Characteristic	Application	Examples
QM	Electronic	High precision; computationally expensive for polymer systems	Calculate the bond energy	[31]
			Calculate the reaction activation energy	[28,32]
RMD	Atomic	Unfixed bond connection; suitable for large polymer systems	Analyze the dynamic changes of species in the aging process	[33,34,35]
			Analyze the reaction path/mechanism	[33,34]
			Calculate the reaction rate	[36]
MD	Molecular	Fixed bond connection; suitable for large polymer systems	Analyze the physical aging of glassy polymers	[37,38,39]
			Analyze changes in polymer properties	[37,40,41,42,43]
			Analyze the physical diffusion process in materials	[44,45,46]

## Data Availability

The data presented in this study are available on request from the corresponding author.
